# The Dangers of Congregate for Children with Diabetes or Other Life-Threatening Medical Conditions

**DOI:** 10.3390/children13010078

**Published:** 2026-01-03

**Authors:** Dennis Michael Styne, Donna M. Petre

**Affiliations:** 1 Pediatric Endocrinology, School of Medicine, University of California, Davis, CA 95616-5270, USA; 2 Superior Court Judge, Judicial Council of California, 455 Golden Gate Avenue, San Francisco, CA 94102-3688, USA

**Keywords:** diabetes, child protective services, foster care, non-related extended family member (NREFM), congregate care facility, The Family First Prevention Services Act (FFPSA), Americans with Disabilities Act, parent mentors for families of children with chronic disease, Novel Interventions in Children’s Healthcare (NICH), Court Appointed Special Advocate (CASA)

## Abstract

Background: Children can be removed from their home if allegations of abuse or neglect are substantiated. The preference is to place them with family members. In the most extreme cases, a child may be placed in a congregate care setting. A child with diabetes should only be placed in such a facility if the staff have been appropriately trained. Otherwise, the consequences can be devastating. In 2022 and 2024, two children were placed into congregate care facilities in Arizona and died of diabetic ketoacidosis due to a lack of appropriate employee training. Study Objective: We aim to inform providers of the legal processes and laws that can result in a child being placed into a congregate care setting. We analyze what went wrong in the care of these two children. We present alternative pathways that might ensure the safety of children before they are placed in such facilities. Methodology: We reviewed public information for cases of morbidity and mortality in children with diabetes in congregate care. We reviewed the California Welfare and Institution legal codes and applicable laws in the Federal Register. We obtained information regarding children with diabetes mellitus who were in the care of child welfare on PubMed. Results and Conclusions: While there are legal safeguards for children with diabetes who are placed in congregate care, these safeguards are ineffective if staff are inappropriately trained. We present programs and recommendations to prevent a child who is placed in a congregate care facility from suffering medical complications or death.

## 1. Introduction

The care of children with diabetes mellitus requires extraordinary conscientiousness by trained caregivers to avoid chronic complications, hospitalizations or death. Ideally, children with diabetes would receive such care in their homes. However, situations arise in which the state intervenes in the life of a child with diabetes and the child is removed from the home. Whether the child is placed with a resource family (previously termed. a foster family) or in congregate care, the child’s safety can only be assured if the caregivers or the staff at the congregate care facility are appropriately trained in the care of the child’s complex disease. If the state fails to ensure that this training occurs before the child with diabetes is placed there, the consequences can be devastating. Unfortunately, in 2022 and 2024, the Arizona Department of Child Safety (DCS) placed two children in two different congregate care facilities who subsequently died of preventable diabetic ketoacidosis due to a lack of appropriate employee training at the facilities. The authors believe that practitioners must be apprised of the legal processes that can be invoked when they suspect a child is experiencing medical neglect. Pediatricians are advocates for the best treatment of children, and providers should know that, despite laws that are in place to protect children tragedies can still occur even when a child is under the care of the state. Thus, we review the legal processes that can lead to a child’s removal from the family home and placement in a resource home or a congregate care facility. We will consider what went wrong in these two cases and offer alternative programs and recommendations to ensure the safety of children with serious health issues, such as diabetes, when the state removes them from their homes.

Primary aim: Inform practitioners of the legal processes involved once they report to a child protection agency that a child is experiencing medical neglect.

Secondary aim: Present two cases of preventable deaths of children due to diabetic ketoacidosis because of a lack of appropriate training of employees in two different congregate care facilities in the State of Arizona.

Tertiary aim: Present alternative programs and recommendations to ensure the safety of children with serious health issues, such as diabetes, before they are placed in congregate care facilities.

## 2. Methods

We present a review article regarding potential complications that can arise when children with complex diseases, such as diabetes mellitus, are placed into congregate care facilities. We suggest programs that may safeguard children from complications in that environment.

We discuss two cases based on public sources of information in local newspapers and broadcast reports. Unless public knowledge, no confidential information relating to these minors is included in this paper. These were the only case reports that we could find from public sources of information regarding deaths of children with diabetes while placed outside of their home under the care of child protective services.

We review the laws related to the placement of a child outside of the home due to medical neglect, focusing on the California Welfare and Institution codes, abbreviated as Cal W & I below. When appropriate, we refer to federal laws. We reviewed appropriate publications in the medical literature in PubMed using such MESH terms as Adolescent, Child, Child Abuse*/mortality, Child Abuse*/prevention & control, Child Protective Services/statistics & numerical data Diabetes Mellitus, Type 1*/psychology, Diabetes Mellitus, Type 1*/therapy, Female, foster care, Glycated Hemoglobin/analysis, group homes (congregate care is not a MESH term), Humans, Male, Adolescent, Behavior Therapy, United States/epidemiology.

## 3. Medical Context

This publication bridges the realms of medicine and law. For readers who are unacquainted with the medical details of the care of diabetes mellitus, we present an introduction to the two cases.

Diabetes mellitus is characterized by the absence of insulin secretion, often partially at the time of diagnosis and completely within months or a few years afterwards. It appears that both children in this review had diabetes for a significant period of time, that they had no insulin secretion at the point of admission to the congregate care facilities and were completely dependent on external insulin sources that had to be administered by their caregiver. Insulin administration will lower a child’s elevated blood sugar value to a normal range from an elevated range that is characteristic of diabetes. Poor blood sugar control due to inappropriate or absent insulin administration will lead to high blood sugar values that can lead to diabetic ketoacidosis (DKA). Diabetic ketoacidosis is characterized by dehydration, usually extremely high blood sugars and an acidic environment in the bloodstream that affects the function of vital body organs and the brain. Diabetic ketoacidosis, if treated with insulin and fluid, can be resolved, and while there may be lingering effects, in most cases, a child can recover. However, more extreme diabetic ketoacidosis can lead to fatal complications, including cerebral edema (swelling of the brain). A child in this advanced state may not recover in spite of appropriate treatment. In sum, insulin administration will usually avoid the development of DKA, and, if appropriately administered in mild or moderate DKA, the child will recover. Absence of insulin at an advanced stage of DKA will be fatal. Diabetic ketoacidosis is avoidable with appropriate insulin treatment.

## 4. Reports of the Two Deaths

On 9 December 2022, the Arizona Department of Child Safety (ADCS) removed a nine-year-old child with diabetes mellitus from his home after his father was arrested on a drug charge [1]. The details of this case are only available from newspaper reports, websites and the ensuing lawsuit. As a juvenile file is confidential, what is known is that the next day, December 10, the child was taken to a (presumably local) hospital.

Within two days, the child was transferred to Phoenix Children’s Hospital, where hospital staff reportedly noted that he was “at high risk of diabetic ketoacidosis (DKA) given his current living situation while in Arizona DCS (Arizona Department of Child Safety) care.” [2].

Two group home administrators at the congregate facility where he was to be placed by DCS on release from Phoenix Children’s Hospital went to the hospital beforehand, where one stated that they received “informal type training” on diabetes over a two-day period by following nursing staff while “they treated [the 9-year-old] and other diabetic patients.” They were given a “booklet” regarding diabetes. Presumably, the hospital staff ensured that the boy received appropriate insulin doses. Five days later, on December 15, the child was discharged to the administrators’ privately contracted congregate care facility. A few days later, the manager of the congregate care facility reported that the child refused to take his insulin, and while the group home stated that it did contact Phoenix Children’s Hospital, there is no available public record stating the results of that contact. There is no information that the congregate care facility spoke with DCS about the child’s unwillingness to take his insulin. The operations manager at the group home was quoted as saying that the policy of this home is “not to force medicate any of the children under their care.” [1]. One day later, on December 20, the child was emergently admitted to Phoenix Children’s Hospital with diabetic ketoacidosis (DKA) for the second time within about 11 days [3]. He died in the hospital on December 26 of DKA and “brain swelling” (presumably cerebral edema) while on a ventilator.

A lawsuit, filed by the father arising out of this case, quotes an internal DCS memo from the director in 2017 stating that before a child is placed in congregate care, “[T]he DCS specialist will make a reasonable effort to identify a grandparent, member of the child’s extended family, or person who has significant relationship with the child who can provide care and meet the child’s needs.” An ABC15 news report from Phoenix states that the child’s grandmother said that she was never contacted for placement [1].

The second case involved the death of a 15-year-old boy admitted to a different privately contracted congregate care facility in Arizona [4]. The mother stated that her child had mental illness and diabetes, and she turned to DCS, because of its experience and facilities for a child with such dual diagnoses. While in the congregate care facility, the boy reportedly refused to take his insulin. A lawsuit brought by the family quotes an email sent from the facility to the DCS approximately one month before his death, stating “[W]e are very concerned that [the 15-year-old] keeps doing this and will result in his death.” A second e-mail, sent weeks before his death from a social worker in the congregate facility to a DCS caseworker, stated: “[H]i team, is there any way to advocate for home nurse for him? It is very alarming they may or may not have appropriate supplies to care for his high medical needs.” A state report documented that before his death, managers in the facility held an emergency child and family team meeting with DCS and concluded that the child was “in need of a higher level of care due to his medical concern and mental health. While the team was in agreeance [SIC agreement], there was no progress on making these changes.” [5]. The administrative rules of the contract between Arizona and the group home stated that a group home must “ensure that each child in care receives all prescribed medication at the prescribed time and in the prescribed dose.”

In July 2024, the facility made a 911 call when an employee reportedly stated, “[Y]es. I have a youth that’s refusing insulin or refusing medical help. Won’t go anywhere. Now he’s making weird noises on the floor,” and later, “He’s making a scene. Now he’s acting like he’s like dead on the floor.” [4,5]. The police first responders arrived to find the boy unconscious on the floor while an employee stated, “[S]o now he’s pretending he’s dead on the floor.” “He’s fine. He’s holding his breath.” He died a few days later on 10 July 2024, in a hospital due to diabetic ketoacidosis.

## 5. Legal Context

### 5.1. The Laws When a Child Is Reported to Be a Victim of Abuse or Neglect

Relying on the laws in the State of California, we will briefly review the laws and procedures of the child welfare system that may lead to a family’s involvement with the department due to medical neglect. The definition of medical neglect is the willful or negligent failure of the parent or guardian to provide a child with medical treatment [6,7]. California mandatory reporters are legally bound to make reports of suspected or known child neglect. These mandatory reporters include care providers in a medical setting, but also teachers, coaches, recreation employees and just about anyone who comes in contact with or is involved in the supervision of children (The Child Abuse and Neglect Reporting Act (CANRA) pursuant to California Penal Code, § 11164 et seq).

Child welfare social workers review the referrals and determine whether a referral requires further investigation. In 2021, the United States reported 3,575,974 cases of possible child abuse, and 16.7% of these cases were substantiated, which means that they were “more likely than not,” true (Cal Penal code section 11165.12.) [8]. The remainder are classified as unfounded or inconclusive. In California in 2023, there were 294,325 cases investigated for abuse, and 17% (50,526) were substantiated [9].

When there are substantiated reports of medical neglect, the Department of Child Welfare Services can provide the family with an informal and voluntary supervision contract that is designed to address the concerns. The parents agree that the social worker will monitor the child’s care to ensure that the child attends doctors’ appointments and that the family follows the medical orders of the medical provider. (Cal W&I 301).

If the social worker determines that court intervention is necessary, a petition will be filed with the juvenile court setting out the specific facts that support a finding of medical neglect (Cal W&I 300). At this hearing, called a detention hearing, the court determines whether the child’s safety requires detention out of the home (Cal W&I 315). If the child is not released to the parent(s), the social worker must assess whether a relative is available for emergency placement. (Cal W&I 309).

After the detention hearing, the court will set another hearing, called a jurisdictional or adjudication hearing, to determine whether the allegations in the petition are true. If the court sustains the allegations as true, the court will make a finding that the child falls under the provisions of California Welfare and Institutions Code section 300 as an abused or neglected child, and the court will assume jurisdiction over the child (Cal W&I 355). If the court asserts jurisdiction over the child, the matter will be set for still another hearing, called a dispositional hearing, at which the court decides what services shall be provided to the family (Cal W&I 358). If the safety of the child permits, the child would remain in the home of the parent(s) under a plan of family maintenance (Cal W&I 16501(g)).

The courts must make reasonable efforts to maintain the child in the family home, unless the safety of the child is at issue. Welf. & Inst. 361(d) However, reasonable efforts are not required if a court determines that one of the three exceptions exists. These are the following:

(1) If the parent has subjected the child to “aggravated circumstances,” as defined by each state, in defining such circumstances, Congress suggested that states include the following: torture, abandonment, chronic abuse, and sexual abuse. (42 U.S.C. Section 671 (a)(15)(D)(i)).
(2) If the parent has committed murder or voluntary manslaughter of another of their children, or felony assault that results in serious bodily harm of any of their children; The exceptions also include aiding, abetting, attempting, conspiring, or soliciting the crime of murder or voluntary manslaughter. (42 U.S.C. Section 671(a)(15)(D)(ii)(III)); The felony assault must cause “serious bodily injury (42 U.S.C. Section 671(a)(15)(D)(ii)(IV)).
(3) Or if the parent’s rights to a sibling of the child have been terminated. (42 U.S.C. Sections 671(a)(15)(A) and (D) and 675 (5)(C)(E)).

If the child is removed from the home, the court will generally provide the parent(s) with family reunification services, the purpose of which is to help the parents reunite with their child in the home (Cal W&I 16501(h)). An individualized case plan would be created that sets out the reasonable services that will be provided to the child and parent(s) to assist them in overcoming the facts and circumstances that necessitated the child’s removal from their care. Cal. W& I 16501.1 These plans, for example, could require attendance at domestic violence and/or parenting classes, individual therapy and drug testing, as well as additional training in the care of a child with diabetes.

The case is reviewed every six months to ascertain the parents’ progress in completing their case plan (Cal W&I 366.21(e) and 366.21 (f)). If an emergency arises, the child welfare department can file a motion under Cal W&I 388 to change a court order if there is a change in circumstances (such as a medical emergency) and it is in the best interest of the child.

Neither California nor the United States denotes medical neglect specifically in their data nor do they specify the type of medical neglect that the reports may allege. This is not unusual, as most states do not have a standardized definition to identify children with complex medical diseases. Because of this, there is an inability to track the care of such children in the child welfare system [10], even though children with medical complexity are more frequently referred for child protective services than children who do not have medically complex cases [11]. A retrospective study of 154 patients reported to child welfare from Lurie Children’s Hospital of Northwestern University stated that 91% of these children had a chronic illness, and the most common diagnosis referred for child welfare investigation was type 1 diabetes [12]. Since diabetes is so often the condition that leads to a report to child protective services, it is important to know how the law views children with diabetes.

### 5.2. How the Law Regards Diabetes

Diabetes is a condition falling under the Americans with Disabilities Act Amendments of 2008 (Americans with Disabilities Amendments Act of 1990, S. 3406 (110th). USA: 2008) of the Equal Employment Opportunity Commission (EEOC). The EEOC specifically amended its ADA regulations, effective 24 May 2011, by adding the impairment of the operation of major bodily functions, including the functioning of the endocrine system, to the list of covered conditions; it is clear that diabetes is covered by this law. The ADA mandates that children with diabetes must have reasonable accommodations for this condition. This law specifically relates to educational and childcare centers but also applies to the child welfare system. The American Diabetes Association sets out clear guidance on the requirements to operate a childcare center if children with diabetes attend [13]. These requirements should likewise be carried into the resource home and congregate care settings. Thus, trained staff members with knowledge of diabetes should always be available if children with diabetes are in the facility.

### 5.3. Laws Regarding Removal of a Child from the Home

The court may place a child outside of a home if parents are alleged to have abused or neglected their child, causing the juvenile dependency courts to take action for the protection of the child (Cal W&I 300). A child may also be removed from the home by state intervention due to juvenile delinquency (Cal W&I 602). When a child is removed from the care of the parents, the state must, in considering where to place the child, give preference to family members if they are interested and suitable to care for the child (kinship care). Federal law (Public Law (P.L.) 96-272 (enacted 17 June 1980) repealed the old foster care provisions of Title IV-A of the Social Security Act, added a new Title IV-E (Foster Care and Adoption Assistance), and amended Title IV-B (Child Welfare Services) of the Social Security Act. 42 U.S.C. Sections 620 *et seq.* and Sections 670 *et seq.* Public Law 105-89; 42 U.S.C. Sections 671(a)(15) and 675 (5)(C)(E)). The Fostering Connections to Success and Increasing Adoptions Act of 2018 requires states to place children with relatives whenever possible and to actively locate and notify them when a child enters the child welfare system. California’s Welfare & Institutions Code Section 361.3 also provides specific requirements to give preferential consideration for placement of children with relatives and non-related extended family members (NREFM) who are willing and appropriate to care for them. A non-related extended family member could be a godparent, coach, teacher or neighbor who knows the child well and who can meet the child’s needs (Cal W&I 361.3). In California when a medically fragile child is removed from the care of the parents, the law seeks to ensure that qualified, medically trained resource parents are considered for placement, while still adhering to existing laws regarding family placement and the child’s overall well-being (Cal W&I § 17739). In cases of children with diabetes or other life-threatening diseases, if the child is placed with a family member or NREFM, it is incumbent upon the social worker to ensure that they have appropriate medical training before the child is placed in their home.

In cases of children with diseases such as diabetes mellitus, the child’s safety can only be assured if the staff of the congregate care facility are appropriately trained in the care of children with this complex disease. If the state fails to ensure that this training occurs before the child is placed in the facility, the consequences can be devastating. Unfortunately, as shown in the cases above, that is what occurred in the State of Arizona when two children were placed by the state welfare authority into two different congregate care facilities and died of preventable diabetic ketoacidosis due to the lack of appropriate staff training at the facilities. To avoid such disastrous results, providers in congregate care facilities must be apprised of and educated in the details of the medical plan for the child, and particularly on the importance of ensuring that the prescribed medications are taken by the child.

We will review how a child may be placed in a congregate care setting, consider what went wrong in these two cases, and offer possible solutions to ensure the safety of children with serious health issues, such as diabetes, before they are placed in congregate care settings.

## 6. Applicable Laws for Children Placed in Congregate Care

Children in only the rarest and most extreme cases should be placed in a congregate care setting (group home). Placement in a congregate care setting should not serve as the long-term living plan for a child [14].

There has been an ongoing concern over the adverse effects of placing children in congregate care. The Family First Prevention Services Act (FFPSA) of February 2018 (enacted as part of Public Law (P.L.) 115–123 as part of the Bipartisan Budget Act) was the federal government’s response to these concerns. The overarching goal of this Act is to direct the child welfare system to avoid family separation wherever possible by the use of in-home support and early preventive services for families at risk. This is a shift in child welfare policy, as the federal government in the past provided funds to support foster care, instead of a focus on maintaining children in their homes. The FFPSA provides federal funding that reimburses up to 50% of the cost to the state and county child welfare systems to keep the child in the home. Since it costs about 7 to 10 times more to have a child placed in congregate care, rather than placed with a family, this funding can represent a significant cost savings [14]. The legislation seeks to reduce reliance on congregate care settings by financially disincentivizing their use. However, if it is determined that the child must be placed in a congregate care setting, the FFPSA includes firm guidelines to make sure that the care is of high quality and provided in accredited facilities with a goal of short-term placement. Further details are provided by the Ann E Casey Foundation (https://www.aecf.org/), which, for decades, has promoted and supported programs to improve the outcomes of disadvantaged youths requiring special care in the court system or in the community and to prevent children from being removed unnecessarily from their homes.

The Family First Prevention Services Act appears to have successfully decreased the number of children in congregate care in the United States from 18% in 2000 to 11% in 2023. In contrast, children in kinship care increased from 25% in 2000 to 31% in 2023 [15,16]. Before a child is placed in a congregate care setting, there must be a comprehensive needs assessment by a Child and Family Team (CFT) (Cal W&I 16501) that addresses the child’s strengths and needs (Welf. & Inst. 11462.04. A Qualified Individual (QI) assessment is also required, which finds that a short-term residential therapeutic program is the setting that will provide the child with the most effective and appropriate level of care in the least restrictive environment. (W & I 4096). A juvenile court review is also required under the FFPSA to assess the child’s needs and determine if they can be met in a less restrictive family-like setting before a child is placed in a residential treatment facility. This process must lead to the development of an individualized case plan for the child that sets out the individualized treatment goals and how the child will be transitioned out of congregate care to a less restrictive environment (Cal W&I 361.2). For children with diabetes, this placement would also require an individualized health plan that includes the following (Cal Code Regs. Tit. 22, § 84069.2):

(A) Documentation by the child’s individualized health care plan team identifying the specialized in-home health care to be administered by one or more health care professionals.
(B) Specific responsibilities of the health care professional(s) for the provision of specialized in-home health care.
(C) Identification of any available and funded medical services that are to be provided to the child in the home, including the name, address and telephone number of each health care professional or agency that is to provide medical services to the child in the home.

## 7. Arizona’s Response to the Deaths of the Children

The Family First Prevention Services Act was in effect in 2018, before the two children died, and should have affected their placement in Arizona before they passed away.

In September 2023, the Arizona Auditor General audited the performance of the DCS and found that “problems related to investigating, taking enforcement action against, and monitoring licensed out-of-home care providers could result in risky or unhealthy environments for children in out of home care.” [17].

In April of 2025, the Auditor General presented a follow-up of the 2023 report that found of the twelve recommendations made to improve the investigation of complaints regarding out-of-home placements, one was resolved and eleven were still in process (see Appendix A for the recommendations and review) [18]. There are plans to again review the progress in 2026.

After the death of the 9-year-old child in 2022, the Arizona legislature reviewed a proposed bill that addressed some of the issues of congregate care, although it is not clear if it was developed in response to the child’s death. Senate Bill 1458, dated 18 April 2024, was sponsored by Arizona State Senator Bennett in a collaboration between Fostering Advocates Arizona, a group of young people with lived experience in foster care, and the Children’s Actions Alliance (Bill Texts: AZ SB1458|2024|Fifty-sixth Legislature 2nd Regular|LegiScan, Home—Children’s Action Alliance). This legislation aimed to reduce the placement of foster children under 12 years old in congregate care settings by requiring the approval of the Department of Child Safety Directors before such a placement is made. Discussion of the Bill noted that Arizona’s rate of congregate care for placement of children under the age of 12, at close to 11%, was the highest in the nation and much higher than the national average of 3%. The requirement of approval of the Department of Child Safety Directors is a nationally recognized best practice supported by the Ann E. Casey Foundation through their Ending The Need for Group Homes project. SB1458 passed the Senate of Arizona with strong bipartisan support, but the Bill failed to pass the House on 4 June 2024. Had it been in effect in 2022, SB1458 would have affected the placement of the 9-year-old with diabetes in congregate care.

The governor of Arizona and a state senator promised accountability for the death of the 15-year-old child with diabetes and said that they would investigate these cases. However, there are no published accounts of the outcomes of such considerations in either of these cases. But the murders of three girls in DCS custody (two likely by strangers and one by family members) who did not have diabetes led to a series of investigative reports by Phoenix ABC television news in September 2025 and a pledge from the governor to investigate foster placement and these deaths in detail [19].

After a meeting involving the general community and tribal leaders (one girl who died was indigenous), Arizona authorities pledged to improve communications between the state foster placement agencies and law enforcement [20]. However, it is not clear that these resolutions address the specific issue of children with chronic diseases, such as diabetes, in congregate care facilities under DCS supervision.

## 8. Clinical Implications

The data from Lurie Children’s Hospital suggests that children with diabetes may frequently be involved in child protective services cases due to medical neglect, but since there are no statewide or national databases that collect this data, it is impossible to know how widespread the problem is [12]. We reported on two Arizona children who died under the care of a state child protection agency while in a congregate care facility, but we do not know how many more children with diabetes or other chronic conditions might have had severe complications while in a congregate care facility or in a foster care placement.

In the case of the 9-year-old boy, the authorities removed the child from the care of the father due to criminal drug charges against him. It is alleged that the department failed to contact available family members, such as the grandmother, who potentially could have served as a caregiver for the child. The federal policy to keep children with relatives in the first instance might have also made a difference if followed. The proposed Arizona law that a child as young as 9 years old would only be placed in congregate care after the specific approval of the Directors of the Department of Child Safety would have provided significant protection for this young child had it passed. The available public records state that the mother of the 15-year-old asked for assistance in view of her son’s mental illness before he was placed in a congregate care setting; it is difficult to ascertain what alternatives were available in his case. Nonetheless, the alleged lack of a timely response by DCS to the concerns of the family and staff at the facility, combined with the lack of education of the staff, led to the tragic result.

While we have emphasized relatives as potential resources to care for a child with a serious disease in foster care, there are reasons that a relative may not be willing to care for a child with medical needs. They may not know enough about the medical condition. It is incumbent upon the state in such cases to provide the education and resources necessary for the relative or the NREFM to properly care for the child.

## 9. What Programs Could Have Avoided Congregate Care Placement?

It goes without saying that the care of a child with a complex disease such as diabetes can be compromised in their own home, as recurrent episodes of diabetic ketoacidosis or other complications of diabetes are certainly found in children living with biological parents, as most experienced practitioners know. Whether the child is in their family home, in kinship care, with a NREFM, or in congregate care, the child’s safety depends on the level of medical education and training that the caregiver or staff have about the disease and their attentiveness to the child’s medical needs. Wherever the child is placed, the caregivers must be appropriately trained to care for the child.

There are resources and model programs available that may prevent children from referrals to child protective services in the first place. The Anne E Casey Foundation produced a report of available federal funding that could provide services to prevent children from entering the child welfare system or placement in foster care. These services include home visits by supportive facilitators, funding families in need with financial support, housing assistance, direct access to medical and mental health treatment programs, substance abuse programs for parents, school-based support, and other sources of support for children and families in fraught circumstances [21]. This report reviews the accomplishments of Colorado, Washington, Massachusetts and North Carolina in leveraging these sources of funding for children and their families in need to improve their situations (more information can be found at www.aecf.org/series/funding-strategies-to-support-families, accessed on 9 May 2025). In the case of the 9-year-old boy, whose father was arrested for allegations of drug use, one of these funding streams for substance abuse treatment might have had some effect on the child’s placement. Since glycemic control in children is associated with parental marital relationship satisfaction [22,23], a funding stream supporting improving family functioning might improve a child’s diabetic control and decrease the likelihood of a child with diabetes entering the child welfare system.

Parents who have successfully cared for their children with chronic diseases have considerable practical wisdom, which can be a source of education and support for families struggling with the care of their children. One such program in Milwaukee, Wisconsin, used parent mentors from urban minority communities to help other parents in the same community who struggle to support their children with asthma. The results demonstrated decreased wheezing, asthma exacerbations, ED visits, and missed parental workdays as the parents demonstrated increased self-efficacy in the care of their children [24]. This one-year randomized controlled study involved monthly visits, telephone calls, and visits from the mentors with the families. The monthly cost of the program was $60.42 per participant, and the expected savings of expenses for medical care for those who actively participated in the program of $46.16 suggests that such a program may be close to self-supporting. A program such as this has potential application to families with diabetes or other chronic diseases and demonstrates the savings that can result and lead to financial support to make it an enduring program.

The same group of investigators studied the success of parent mentors in enrolling minority families who qualified for insurance coverage but lacked it [25]. Absence of insurance is a significant stress on parents who cannot obtain professional advice for the care of their children and can result in contact with the child welfare department. The investigators held a randomized controlled trial using parent mentors in the intervention arm and found significantly more success in obtaining insurance coverage for children compared to traditional methods of obtaining insurance (95% vs. 68%), as well as successful coverage renewal (85% vs. 60%). Families with parent mentors were more likely to have a primary care physician (39% vs. 15%) and had less difficulty in obtaining specialty care (46% vs. 11%). Two years after parent mentor cessation, more children whose parents had parent mentor support were still insured (100% vs. 76%). The parent mentor program saved $6045.22 per year per child insured but cost $636.60 per child per year, suggesting that a program like this can be self-sustaining [26]. A recent scoping review of peer mentorship in programs for children with chronic diseases sets out the aspects of these programs that are more likely to achieve success. They include flexibility in the program structure, the inclusion of knowledgeable and dedicated mentors, adequate support for the mentors, and, of course, support for the financial requirements to continue the program [27]. Based upon this data, peer-to-peer mentorships for families of children with diabetes may also hold promise for improving the care of these children.

Another program that has shown success at three institutions (Oregon Health and Science University, Stanford University and University of California, San Francisco) in improving the care of children with diabetes, who might have otherwise been referred to child protective services, is the Novel Interventions in Children’s Healthcare (NICH). NICH was developed to improve the care of children with chronic disease in an environment with a high degree of social risk. Diabetes mellitus was the focus of the initial trial [28]. One aspect of NICH is delivering Behavioral Family Systems Therapy (BFST) to enrolled families with a focus on problem-solving, communication skills training, cognitive restructuring, and family systems interventions to identify and remedy dysfunctional family interactions that may affect the caregivers’ ability to provide optimal care for children with diabetes.

NICH uses master-level interventionists, trained in the basics of diabetes (but not to the level of a medical provider), to achieve care coordination between medical providers and the child and family to enhance communication, adherence, and solve issues interfering with the treatment regimen. NICH interventionists also provide case management by interacting with schools, community agencies, child protective services, and the other agencies that may support the family (e.g., juvenile office personnel, employers), as well as any other organizations that may provide useful resources. The interventionist works in the child’s home environment, community and school. While they are not social workers or medical providers, they can interact with social services, caregivers or employers with an overall goal of solving some of the daily problems of the family that may interfere with the care of the child. They may try to address transportation difficulties to ensure that the patient reaches clinic appointments, they may attend medical appointments to provide support for the family, or they can help obtain already ordered medical supplies when roadblocks occur.

NICH was introduced into a population of historically marginalized, racially and ethnically diverse families with high social stress who had children with diabetes and proved to bring about a significant mean hemoglobin A1C reduction of −1.1% and an increase in the use of continuous glucose monitoring (27% to 73%) at 1 year following enrollment. In addition, there were significantly fewer hospital admission days, significant reductions in depressive symptoms in patients, and improvement in the diabetes distress of the caregivers [29]. The savings resulting from the decrease in hospital days is a form of information that might be used to convince medical centers, government funding agencies and insurance companies to support the institution of this program in other locations.

The Court Appointed Special Advocate (CASA) program involves adult volunteers who are appointed to assist a child who is already involved in the welfare system and serve as advocates for the child [30]. These volunteers engage with children whether they remain in their home, are placed with relatives, NREFM, resource families, or in congregate care facilities. They evaluate their situations and if they find concerns, they can confer with the child’s lawyer, the social worker or the court to try to improve their care. This program is not set up for medical supervision of a child with a complex disease like diabetes, but the volunteers come from all types of work, including medical professionals. They may, but are not required to, be involved in monitoring the child’s medical care. However, if they find a problem, they may report it.

Apart from these programs, we suggest that medical providers for children with complex conditions receive notice before a child under their care is removed from the family and placed in a congregate care setting. They must also be involved in the creation of an individualized medical treatment plan for the child, and the facility must receive a copy of the plan and contact information for the provider in case of emergencies. Mandatory uniform training guidelines for each life-threatening medical condition should be created for training staff in congregate care facilities (Table 1 and Table 2).

## 10. How Does Europe Address Child Welfare Issues?

A detailed evaluation of child welfare practices throughout the world is beyond the scope of this paper. However, we note that Europe, in general, tends to discourage congregate care when a child is removed from the home. In 2021, European laws and policies emphasized shifting from institutional congregate care to family-based solutions for children, driven by the EU Strategy on the Rights of the Child and the European Child Guarantee [31], focusing on individual assessments and family-like settings over large institutions. Key legal frameworks promote supporting children within their families or in smaller, family-type foster care and prevent separation where possible. UNICEF published a white paper on the development of foster care in Europe and Central Asia in 2024, focusing on “reduced reliance on harmful institutional care.” [32]. Instead, they suggest new community-based services for family support in cases requiring alternative care. They point out that kinship care is the best alternative to parental care, but note that it is becoming more difficult to find kinship caregivers due to migration and economic conditions, and there is a limitation of system capacity to support it. As a result, many countries included in the report must rely on congregate care (they denote it as institutional care) as the only answer when a child is removed from the home.

## 11. Discussion

To our knowledge, no one has addressed the deaths of children with diabetes in congregate care in the medical literature. The goal of the authors is to fill a gap in the basic legal knowledge of children’s medical providers when they consider reporting cases of medical neglect and the dangers of placement of their patients who have medically complex conditions in congregate care. Reliance upon publicly available news stories and lawsuits clearly limits the scope of this publication and presents a weakness. Likewise, we limited the discussion to the laws of California and some federal statutes. We understand that the other states in the United States generally approach child welfare in a similar manner to California, but the scope of this paper does not allow for an exhaustive review of the processes in other states. The laws of the State of Arizona are referenced only as they apply to the children who died in DCS’s care.

The authors admit certain biases. The authors believe that decisions regarding the medical care of children should rest with trained medical providers, particularly when a child with a complex medical disease is removed from the home by the court and placed in a congregate care facility. The aim of this publication, however, is to bring attention to the problems children with complex diseases might experience in congregate care facilities, hoping that further discussions will lead to improvements that would prevent more deaths.

## 12. Conclusions

The care of children with chronic diseases such as diabetes is always challenging, but when a child is removed from the home and placed in a congregate care setting, the dangers may increase dramatically. At this time, there is no data available to inform us just how often other deaths have occurred in congregate care settings, or any other foster care settings, for children with diabetes or how many “close calls” have occurred involving children with diabetes or other serious diseases that have led to serious complications short of death.

We use these two case reports to focus the reader on the laws and procedures in the juvenile courts that control cases involving medical neglect of a child. We believe this information is not within the general knowledge of providers. We point out safeguards that are supposed to be in place for such a child, but which are not always implemented. We suggest policies and programs that might prevent such deadly outcomes. Surely other innovative programs can be developed to safeguard a child with chronic diseases who is placed in congregate care. It is a tragedy when any child dies, but it is all the more tragic if it is preventable, and even more so if it occurs when the child is under the care of the state.

## Figures and Tables

**Table 1:** Programs to avoid or improve congregate care placement.

Programs That Aim to Avoid Congregate Care Placement	Requirements for Implementation
Kinship care	Potential family caregivers must be contacted and, if interested, evaluated for appropriateness of placing a child in their care
NREFM care	Potential caregivers must be contacted and, if interested, evaluated for appropriateness of placing a child in their care
Resources found in the Ann E. Casey Foundation’s: Key funding streams to keep families supported, connected and safe.	Programs are available in individual states and counties and the federal government to address problems in the child’s home
Parent mentor programs	This promising program proven in asthma holds potential for diabetes but must be set up locally with adequate resources
Novel Interventions in Children’s Healthcare (NICH). NICH	This proven program for diabetes must be set up locally with adequate resources
Adherence to existing guidelines and law	Failed in the two cases presented; procedures and laws must be implemented

**Table 2:** Programs to provide oversight if a child is placed in congregate care.

Programs to Provide Oversight If a Child Is Placed in Congregate Care	Requirements for Implementation
Court Appointed Special Advocate (CASA)	Not aimed to prevent placement but allows placement to be monitored for any overall concerns including medical needs, particularly if the CASA has a medical background
Childs medical provider oversight after placement	Provider is rarely included in oversight once patient is placed but there should be a procedure established to make the provider an integral part of the child’s medical care while in placement
Mandatory uniform training guidelines for each life-threatening medical condition should be created for training staff in congregate care facilities	Development of the guidelines and establishing the training

## Data Availability

No new data were created or analyzed in this study.

## Appendix A

Auditor General Review State of Arizona 2025

1. Further develop and implement its draft licensing complaint-handling procedures to include time frames for each key foster home and/or group home licensing complaint investigation and enforcement step, including time frames for assigning licensing complaints for investigation and taking action in response to validated licensing complaints, and complete licensing complaint investigations and take enforcement actions consistent with these time frames.

Status: Implementation in process.

2. Further revise and implement its draft guidance for taking a risk-based approach to prioritize foster home and group home licensing complaint investigations by specifying which types of allegations correspond to each prioritization level, including licensing complaint investigations opened in response to abuse/neglect allegations related to child welfare agency staff, staff requirements for documenting the prioritization level, and what actions/activities staff should take to initiate an investigation.

Status: Implementation in process.

3. Further revise and/or develop procedures for interviewing staff and children residing at foster homes and group homes during licensing complaint investigations, including guidance for determining when children should or should not be interviewed.

Status: Implementation in process.

4. Develop and implement written guidance for staff to work with law enforcement when conducting licensing complaint investigations opened in response to abuse/neglect allegations related to child welfare agency staff, including how its staff should work with law enforcement to share information and/or coordinate licensing complaint investigations with law enforcement personnel and when and how its staff should review the results of law enforcement investigations.

Status: Implemented at 18 months

5. Revise and/or develop and implement written guidance for staff to research foster home and group home licensee violation history.

Status: Implementation in process.

6. Revise and/or adopt new rules for child welfare agency licensing complaint handling, as necessary, to authorize a greater range of enforcement actions.

Status: Not implemented.

7. Consistent with the Department’s rules revised in recommendation 6, update and implement the Department’s graduated system of enforcement actions for validated child welfare agency/group home licensing complaints and include guidance for staff specifying the violations that would lead to different enforcement actions, including mitigating and/or aggravating factors staff should consider.

Status: Implementation in process.

8. Develop and implement a graduated system of enforcement actions for validated foster home licensing complaints and include guidance for staff specifying the violations that would lead to different enforcement actions, including mitigating and/or aggravating factors staff should consider.

Status: Implementation in process.

9. Further develop and implement policies and procedures regarding ongoing monitoring of group homes, including assigning staff responsibility for conducting ongoing monitoring, outlining how to select facilities for monitoring and complete the site visits checklist, and specifying the frequency of site visits and providing guidance for risk-based and unannounced site visits; and perform ongoing monitoring consistent with the policies and procedures.

Status: Implementation in process.

10. Add data fields to Guardian and/or another IT system for key dates in the licensing complaint-handling process, including the investigation start date, the investigation completion date, and the enforcement action date.

Status: Implementation in process.

11. Develop and implement a method in Guardian and/or another IT system to combine multiple licensing complaints it receives for the same licensee into the same licensing complaint entry, including combining relevant details from each entry; and develop monitoring reports to keep track of these licensing complaints that have been combined.

Status: Not implemented.

12. Require tracking, supervisory review, and managerial oversight of the licensing complaint investigation and enforcement processes and regular ongoing group home monitoring to verify staff compliance with Department policies, procedures, and time frames. Add reporting capabilities to Guardian and/or another IT system, as necessary, to help Department staff track, review, and oversee these processes.

Status: Implementation in process.
